# Effect of Training Combining Hand-Arm Bimanual Intensive Therapy Concept and Postural Control Elements on Individuals With Chronic Incomplete Cervical Spinal Cord Injury: A Report of Two Cases

**DOI:** 10.7759/cureus.79695

**Published:** 2025-02-26

**Authors:** Kazumasa Jimbo, Takashi Murayama, Kousuke Takahama, Tomohiro Yoshimura, Takuya Goto, Tomonari Tosaka, Kentaro Suzuki, Masahiko Kitagou, Naohisa Kikuchi

**Affiliations:** 1 Department of Rehabilitation Treatment, Chiba Rehabilitation Center, Chiba, JPN; 2 Department of Rehabilitation Medicine, Chiba Rehabilitation Center, Chiba, JPN

**Keywords:** cervical spinal cord injury, intensive training, rehabilitation, task-oriented training, upper limb function

## Abstract

The complex nature of tetraplegia in individuals with incomplete cervical spinal cord injury (ICSCI) significantly impacts activities of daily living, with few interventions comprehensively addressing upper and lower limb and trunk dysfunction due to tetraplegia. This study aimed to investigate the effectiveness of intensive task-oriented training incorporating bimanual movement and postural control in individuals with ICSCI. This study included two cases: a man in his 30s (neurological level of injury (NLI) C5; American Spinal Injury Association Impairment Scale (AIS) grade D) and a man in his 50s (NLI C3; AIS grade D). The intervention consisted of 50-h task-oriented training over 15 days and a diary-based transfer package. Both cases demonstrated improvements in upper limb function and balance ability. Several goals were improved or achieved. This report indicates the efficacy of intensive task-oriented training for ICSCI. Bimanual activities in various postures, including standing, enhanced upper limb function, and balance ability. This indicates that interventions targeting upper limb function in ICSCI should consider both bimanual movement and postural control. This study highlights the ability of this comprehensive training to improve functional outcomes in individuals with ICSCI and provides valuable insights into spinal cord injury rehabilitation.

## Introduction

Recently, the number of individuals with cervical spinal cord injury (CSCI) has increased in various regions, with a particularly large number of individuals with incomplete cervical spinal cord injury (ICSCI) [1,2]. Upper limb dysfunction due to CSCI significantly impacts activities of daily living (ADLs) [3,4]. Furthermore, factors such as upper limb function and balance ability in CSCI have been reported to affect self-care [5]. Various interventions have been developed for upper limb dysfunction in ICSCI [6]. However, ICSCI has various symptoms [7], making the development of a standardized intervention that addresses bimanual upper limb dysfunction due to tetraplegia and posture control, including lower limb and trunk function, challenging.

Hand-arm bimanual intensive therapy (HABIT) and hand-arm bimanual intensive therapy including lower extremities (HABIT-ILE) have proven effective for cerebral palsy, a type of tetraplegia similar to ICSCI [8-10]. HABIT is an intensive task-oriented training method focusing on bilateral upper limb activity based on the concept of constraint-induced movement therapy (CIMT) [9]. HABIT-ILE is an intervention that adds a postural control perspective to bimanual movement training similar to HABIT, and many studies have been conducted on it in recent years [11,12]. These interventions are based on a motor learning approach and involve high-intensity tasks of adjusting difficulty to meet specific goals: for example, participants might undergo 50 hours of training over 10 days or 90 hours of training over 15 days [9,12]. Furthermore, the effectiveness of bimanual movement in stroke has been verified. Practicing ADLs or similar tasks with bilateral upper limbs improves the coordination of the upper limbs and the control of bilateral hands and is more effective than conventional interventions [13-15].

These intervention perspectives can be applied to individuals with ICSCI with central nervous system disorders involving bilateral upper or lower limb and trunk dysfunction. However, reports on intensive task-oriented training that incorporates bimanual movement and postural control in ICSCI are limited, and practical reports are needed. Therefore, this study aimed to examine the intervention content and effects of intensive training (HABIT for spinal cord injury (HABIT-SCI)) that applies the concept of bimanual movement training, such as HABIT and HABIT-ILE, for cerebral palsy or stroke from previous studies to chronic ICSCI. The practical content of this study will be beneficial when implementing intensive upper limb functional training for ICSCI in the future. Additionally, it has the potential to broaden the range of intervention methods for ICSCI.

This article was previously presented as a meeting abstract at the 59th Annual Meeting of the Japan Medical Society of Spinal Cord Lesion on November 7-8, 2024.

## Case presentation

Case 1

Case 1 was a 30-year-old man (neurological level of injury (NLI) C5; American Spinal Injury Association Impairment Scale (AIS) grade D). He was injured in a motorcycle traffic accident. This case was admitted to a rehabilitation center specializing in spinal cord injuries 23 days post-injury and underwent rehabilitation. At admission, he presented with NLI C5 and AIS grade D. According to the International Standards for Neurological Classification of Spinal Cord Injury, the Upper Extremity Motor Score (UEMS) was 46 points, the Lower Extremity Motor Score (LEMS) was 50 points, and the sensations of both lower limbs were severely impaired. He could perform ADLs using a wheelchair, special environment settings, and assistive devices, and partial assistance was required. Rehabilitation included walking training with a walker. The goals set for implementing HABIT-SCI were “writing,” “putting on pants while standing,” “cleaning one’s ears,” “cutting one’s nails,” “getting up and moving on the floor using one’s hands,” “opening a package,” “wiping one’s bottom and inserting a suppository,” “washing one’s armpits,” “double-click on a computer,” and “button one’s clothes.” HABIT-SCI was conducted 255 days after injury.

Case 2

Case 2 was a 50-year-old man (NLI C3; AIS grade D). He was injured in a bicycle racing accident. This case was admitted to a rehabilitation center specializing in spinal cord injuries 32 days post-injury and underwent rehabilitation. At admission, he presented with NLI C3 and AIS grade D. The UEMS was 33 points, and the LEMS was 31 points. He could perform ADLs using a wheelchair, a Lofstrand crutch, special environmental settings, and assistive devices. The goals set for implementing HABIT-SCI were “taking things off the top while standing up,” “standing up from the floor,” “closing one’s armpits when performing an action,” “using fingers and wrists when writing,” and “turning coins around and inserting them into a vending machine.” HABIT-SCI was conducted 527 days after the injury.

Intervention

**Figure 1:**
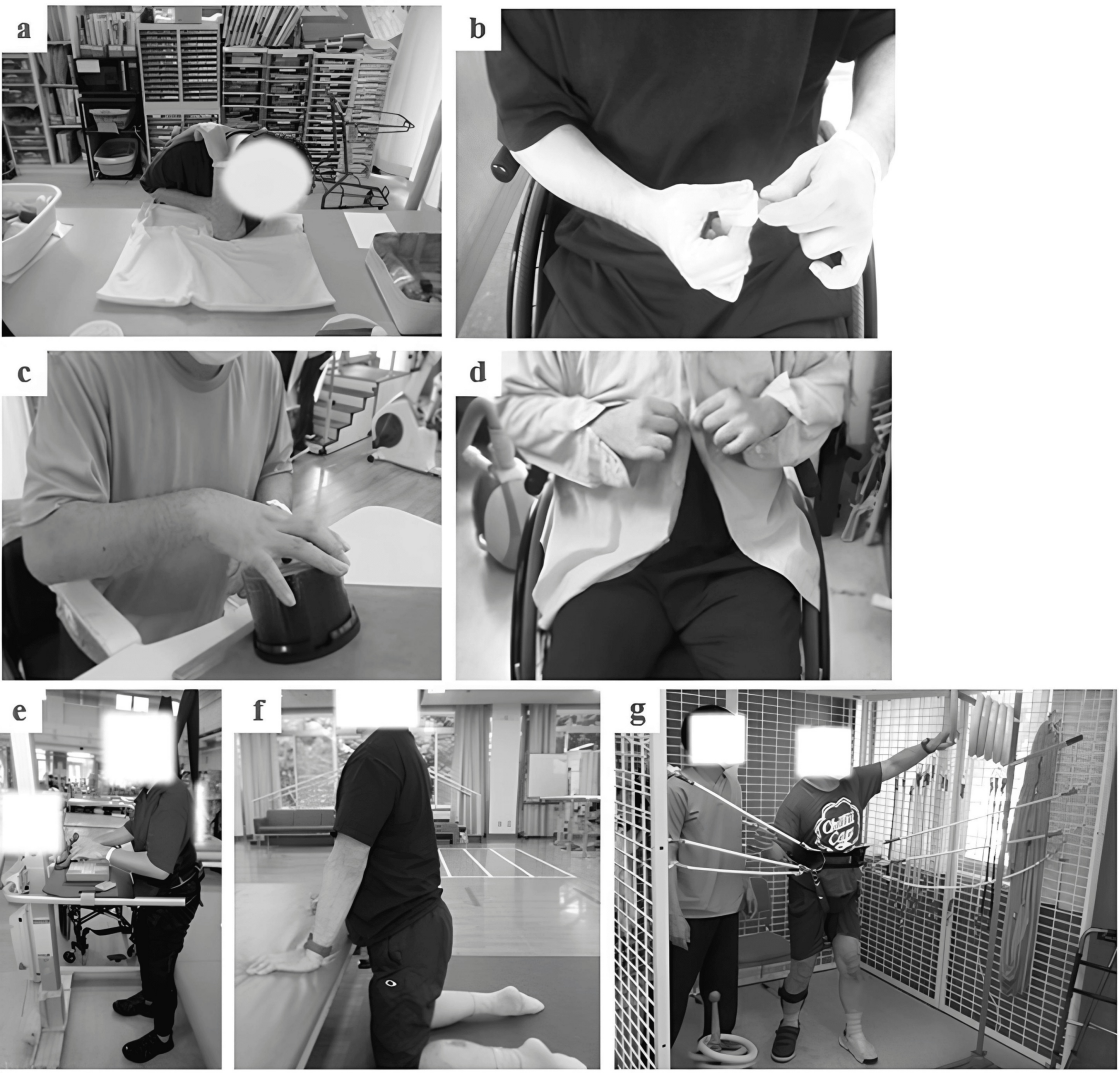
Training examples (a) Exercises to increase the range of motion of the shoulder joint. (b) Pinch strength training. (c) Finger extension training. (d) Button one's clothes training. (e) Static upper limb functional training in a standing position using a body weight support device. (f) Floor-based movement training. (g) Dynamic upper limb functional training in standing using a body weight support device Examples of upper limb functional training. a-c show self-training with the goal activity movement elements divided into several parts. d shows the actual goal activity practice. e shows the use of a body weight support device to safely enable the self-training of upper limb activities in the standing position. This setting allowed for safe self-training for long periods of time. f shows self-training to improve postural control on the floor, including many movements that involve supporting body weight with the upper limbs, such as crawling. g shows the use of a body weight support device to perform more dynamic upper limb movements by reaching in different directions. Case 1 performed this as part of practicing changing underwear while standing. Case 2 performed this as part of training to reach for an object on a shelf.

HABIT-SCI was conducted with reference to the HABIT and HABIT-ILE and bilateral arm training protocols for stroke [9,12,14]. Before starting HABIT-SCI, the goals were shared with the case, the elements of those goals were extracted, and tasks for bimanual, standing, and floor movements were set. For standing movements, activities involving bimanual movement using a body weight support device were included (Figure 1). Applying bilateral arm training for a total treatment time of 30 h or more has been reported to increase positive results [14].

**Figure 2:**
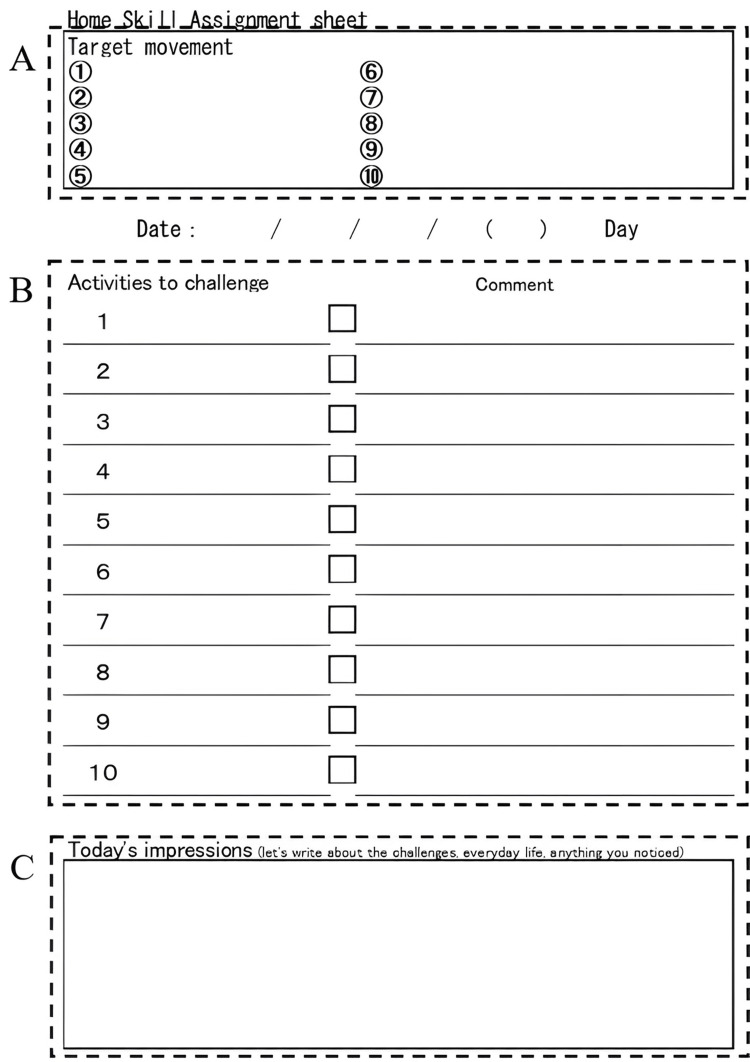
Transfer package This is a diary for reflecting on your goals every day. (A) The goal of performing HABIT-SCI was written down. (B) Activities to challenge. The cases wrote down the goal for the day every morning. In the Comment section, the cases provided self-feedback on the progress of the goal every night. (C) The cases wrote down the condition and impression of the day every night.

Therefore, the intervention consisted of 3-4 h of self-practice per day and training with occupational therapists (OT) and physical therapists (PT) and was conducted for a total of 50 h over 15 days. Additionally, a transfer package (TP) was introduced to the cases, in which the cases were asked to reflect on their daily tasks and record the status of their goals in a diary (Figure 2).

**Figure 3:**
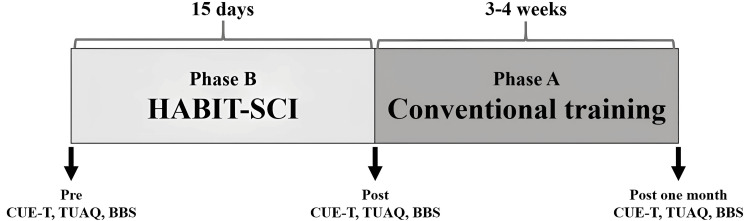
Intervention flow of this report This figure outlines the intervention and evaluation process in this report. HABIT-SCI: Hand-Arm Bimanual Intensive Therapy for spinal cord injury; CUE-T: Capabilities of Upper Extremity Test; TUAQ: Tetraplegia Upper Limb Activities Questionnaire; BBS: Berg Balance Scale

Methods were devised to set tasks for self-practice, and the target goals were divided into 10-20 tasks. Tasks could be updated as needed based on improvements in the target goals. Furthermore, the TP was used every day to set and review goals, update goal movements, and strive to maintain motivation. The two cases filled out the TP every day, and the OT provided feedback on the contents. The effectiveness of HABIT-SCI was examined using a single-case BA design. After the 15-day HABIT-SCI (phase B), the conventional training period (phase A) was held for 3-4 weeks, during which OT and PT training, computer practice, and ADL practice were performed for 1-2 h per day. Upper limb function and balance were evaluated before and after each period (Figure 3).

In Case 1, a combination of functional electrical stimulation therapy, repetitive transcranial magnetic stimulation, and robotic therapy was used to improve upper limb function in phases A and B. In both cases, conventional interventions similar to phase A were administered before HABIT-SCI.

Outcome measures

HABIT-SCI consists of interventions that focus on bimanual movement and balance ability. Therefore, an evaluation specialized for CSCI was selected, encompassing a wide range of perspectives, including bimanual movement evaluation and balance ability evaluation. Additionally, HABIT-SCI includes a strong element of task-oriented training. Therefore, an evaluation composed of subjective elements of the case regarding upper limb function and ADLs was also performed.

Upper Limb Function Evaluation

Capabilities of upper extremity test (CUE-T): The CUE-T was developed by Thomas Jefferson University in the United States to evaluate upper limb function in CSCI. The CUE-T demonstrated excellent reliability, validity, and responsiveness [16]. The CUE-T consists of 15 unilateral (tested separately on the right and left sides) and two bilateral items (32 items in total), including bimanual, gross, and skillful movements, allowing for a more detailed evaluation of upper limb function. The minimal important change (MIC), severity classification, and cutoff values ​​for ADL independence are shown in previous studies [17,18]. The anchor method was used for this MIC. The MIC for subacute CSCI (within nine months of injury) in data collected at one-month intervals was 7.7 points (hand: 2.0 points, side: 3.7 points) [18].

Tetraplegia Upper Limb Activities Questionnaire (TUAQ)

The TUAQ is a patient-reported outcome of upper limb activity in CSCI. The TUAQ comprises 10 items based on the International Classification of Functioning, Disability and Health. Items are subjectively scored by the subject on a 5-point scale based on the Canadian Occupational Performance Measure criteria: “Performance: 1 = not able to perform and 5 = able to perform extremely well” and “Satisfaction: 1 = not satisfied with the performance at all and 5 = extremely satisfied“ [19,20].

Balance Ability Evaluation

Berg Balance Scale (BBS): The BBS was developed to assess balance ability in older adults and stroke patients and monitor changes in balance ability over time [21]. The BBS comprises 14 items that assess postural stability, such as static and dynamic balance. Each item is scored on a 5-point scale, ranging from 0 (unable to perform the task) to 4 (best performance). The total score ranges from 0 to 56 points. The BBS is used to predict falls in various diseases. The BBS demonstrated high reliability and validity in SCI [22]. The anchor method was used to determine the MIC for BBS and for patients with balance disorders was 7.0 points [23].

Results

Case 1

**Figure 4:**
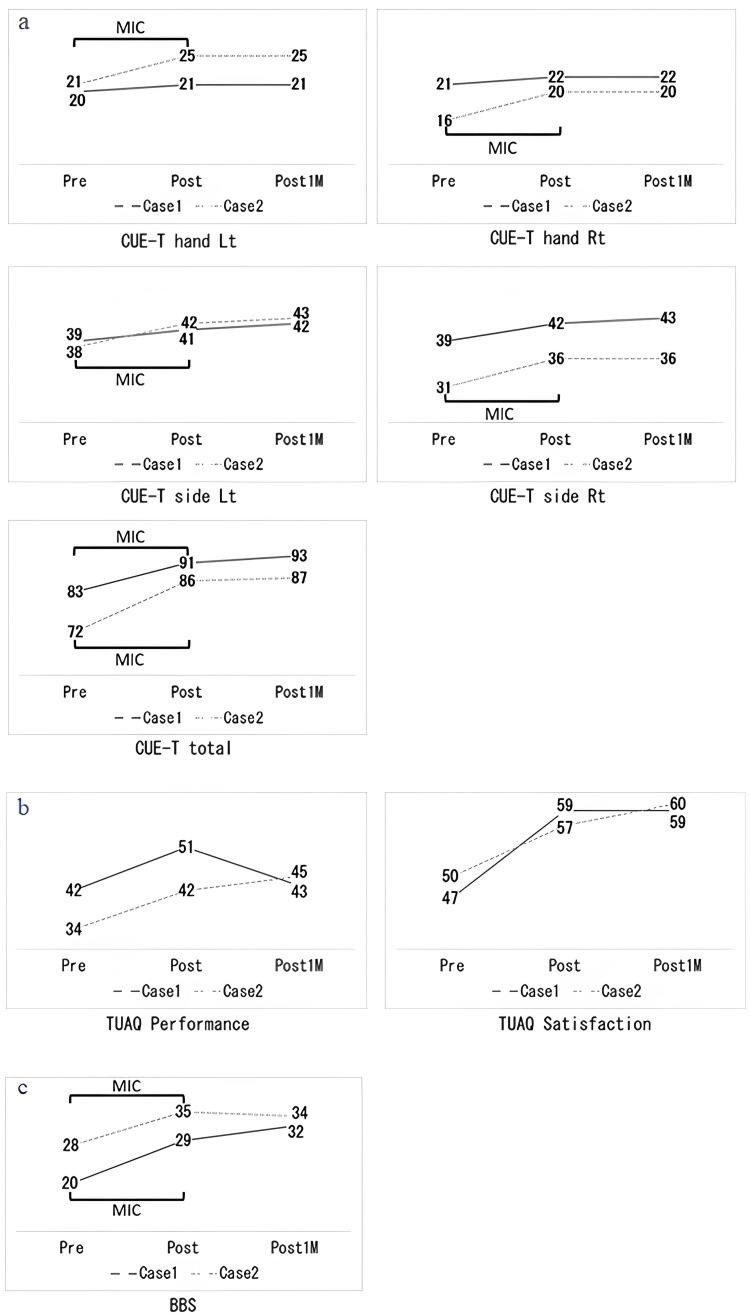
Changes in upper limb function and balance function evaluations (a) The results of the CUE-T. (b) The results of TUAQ. (c) The results of BBS. MIC: minimal important change; Rt: Right; Lt: Left; Post1M: Post one month; CUE-T: Capabilities of Upper Extremity Test; TUAQ: Tetraplegia Upper Limb Activities Questionnaire; BBS: Berg Balance Scale

In phase B, the CUE-T total and BBS scores showed changes that reached the MIC (CUE-T = 7.7 points [18]; BBS = 7.0 points [23]) (Figure 4).

The TUAQ score improved in phase B. In phase A, satisfaction did not change, and performance decreased. The case showed improvement in the degree of independence and satisfaction in movements. The goals including “writing,” “putting on pants while standing,” “cleaning one’s ears,” “cutting one’s nails,” “getting up and moving on the floor using one’s hands,” “opening a package,” “wiping one’s bottom,” “washing one’s armpits,” “double-click on a computer,” and “button one’s clothes” were achieved.

Case 2

In phase B, the CUE-T hand and side and BBS scores showed changes that reached the MIC (CUE-T hand = 2.0 points; CUE-T side = 3.7 points [18]) (Figure 4). Furthermore, the CUE-T and TUAQ scores improved slightly, even in phase A (Figure 4). The case showed improvement in the degree of independence and satisfaction in movements. The goals including “taking things off the top while standing up,” “closing one’s armpits when performing an action,” “using fingers and wrists when writing,” and “turning coins around and inserting them into a vending machine” were achieved.

No adverse events were observed in either case during phase A or phase B.

## Discussion

In this study, the concepts of HABIT and HABIT-ILE and bilateral arm training protocols for stroke from previous studies were applied to two cases of ICSCI, and intensive training (HABIT-SCI) was conducted that focused on bimanual movement and postural control. The cases were in the chronic phase more than six months after injury. However, when HABIT-SCI was performed, the CUE-T, which comprehensively evaluates upper limb performance, and the BBS, which evaluates balance, showed improvement, reaching the MIC. Additionally, both cases demonstrated improvement and achieved many of the goals. Therefore, short-term intensive task-oriented training incorporating the perspectives of bimanual movement and postural control and the introduction of TP may be an effective intervention for ICSCI.

In both cases, the CUE-T scores significantly improved, indicating improvement in gross, skillful, and bimanual movements. Notably, both cases showed improvement in the CUE-T items “Lift Up,” which involves lifting weights with both upper limbs, and ”Push Down,” which involves push-up movements. These improvements may be due to the use of the upper limbs as support, as the tasks included activities related to postural control, such as balance movements in the standing position and floor-based movements. Case 1 could crawl and kneel independently after HABIT-SCI, indicating not only improved trunk stability but also enhanced ability to support body weight with the upper limbs. Functional impairments of the upper limbs and trunk have been reported to significantly affect activities in ICSCI [4]. A previous study reported that activities on the floor or in a standing position with SCI affect other activities, such as walking [24], which highlights the importance of incorporating postural control into upper limb activities as well. Additionally, in Case 2, the CUE-T hand score reached the MIC, indicating that HABIT-SCI helps improve not only gross movements but also skillful movements.

Regarding upper limb function, both cases showed improvement in the TUAQ score in phase B. Case 2 showed improvement even in phase A. These findings indicate that a goal-focused lifestyle is associated with improved functional ability even after HABIT-SCI. However, Case 1 showed a decline in performance during phase A. This may be due to the significant improvement in function and goals during HABIT-SCI.

HABIT-SCI sets multiple goals at the start. Some goals were improved or achieved in both cases. Intensive training focusing on bimanual movement has been reported to be more effective than conventional interventions in stroke. Furthermore, interventions focusing on bimanual movement are superior to CIMT in approaching ADLs [13-15]. Individuals with ICSCI have tetraplegia, and training with only one hand is unlikely to improve activities. Therefore, incorporating the perspective of bilateral movement into the ICSCI intervention is necessary.

In this study, both cases showed improvement in the BBS score, reaching the MIC, and the range of activities in the standing position expanded. Case 1 was able to change underwear while standing, and Case 2 was able to easily reach objects above while standing, which led to improvements in real-life activities. These findings highlight the importance of combining the perspectives of upper limb activity and postural control, such as standing, when focusing on activities in ICSCI.

The TP, through a diary, helped both cases reflect on their goals. Case 2 was able to live a life conscious of his goals even after HABIT-SCI, which led to the maintenance and improvement of upper limb function and balance function even in phase A. The TP is an effective method to enable the use of the paralyzed hand in daily life as a concept of CIMT for stroke [25]. Although this study focused on ICSCI, a condition characterized by bilateral paralysis, we believe that the TP is an effective method of connecting participants to activities by supporting them in thinking about problems and incorporating them into their daily lives.

In addition, one of the key components of this study is long-term self-practice and management of motivation. Setting and updating assignments were important for these elements. We believe that the use of a daily TP and feedback to review tasks and goals, and the regular updating of self-practice tasks, contributed to maintaining the motivation of the case. In recent years, remote rehabilitation at home after discharge from the hospital has become a hot topic, and its necessity has become an important perspective in the field of ICSCI [26]. Therefore, self-practice-based task-oriented training such as that in this study may contribute to the diversification of ICSCI rehabilitation in the future.

On the other hand, some goals could not be achieved, such as “inserting a suppository” in Case 1 and “standing up from the floor” in Case 2. These goals were difficult, and the initial goal-setting method is a future issue. Furthermore, the two cases to which HABIT-SCI was applied had upper limb function at the moderate-to-mild level based on the CUE-T [17] and were able to stand and walk. Therefore, interventions for individuals with milder and more severe conditions are necessary. Both cases showed clinically meaningful changes despite being in the chronic phase. This study focused on performance and patient-reported outcomes. Further studies with a larger number of cases and neurological and physiological evaluations are needed to investigate the effects and mechanisms of improvement in more detail.

## Conclusions

The effectiveness of intensive task-oriented training for various central nervous system diseases and bilateral arm training protocols for stroke was demonstrated. This study applied these concepts and demonstrated the efficacy of short-term task-oriented intensive training focusing on bimanual movement and postural control for chronic ICSCI. These findings may provide a new perspective for standardizing rehabilitation for ICSCI. Future research should focus on verifying goal-setting methods, evaluation content, and intervention protocols. Furthermore, it is essential to consider developing a system that is more effective for CSCI and applicable across various rehabilitation settings.
